# Trephining in Its Ancient and Modern Aspect

**Published:** 1894-12

**Authors:** 


*Trephining in its Ancient and Modern Aspect.* By JOHN FLETCHER HORNE, M.D. Pp. xi., 133. London: JOHN BALE & SONS. 1894.

We have in this book a very interesting historical account of the operation of trephining extending from the neolithic period down to the present day. The author acknowledges his indebtedness to M. Broca for much of the early literature of the subject, and he has also drawn freely upon other writers, both European and American, so that only a small part of the book can be called original. He has unfortunately forgotten sometimes to acknowledge the origin of his information, for Dr. J. B. Hamilton points out [1] that long extracts have been freely taken from an address delivered by him at Detroit in 1892 and published in the same year. [2]

The latter part of the book is devoted to the modern surgery of the skull; but we cannot congratulate the author on his treatment of this portion of the subject. Four successful cases are reported as having come under his notice, either under his immediate care or that of his colleagues; but although the

[1] *J. Am. M. Ass.*, vol. xxii., 1894, p. 234. [2] *Ibid*, vol. xviii., 1892, p. 733.

result is described as being in each case most satisfactory, yet we observe that two cases out of the four were followed by free suppuration, and in one a hernia cerebri had to be dealt with before the wound healed. The important subject of trephining for intra-cranial suppuration the result of ear disease is dealt with in a single paragraph of eighteen lines, and no mention whatever is made of sinus thrombosis.

It is a pity that the author did not obtain the assistance of a friend to read the proofs for him. The errors of spelling, especially in proper names, are much too numerous for the book to be a credit: instances are to be found in the introduction, and on pages 7, 8, 18, 43, 47, 79, 104, &c. The book also deserves censure for the inexcusable absence of an index.

